# Reply to Mattei et al. Jacob Disease, Osteochondroma of the Coronoid Process, Coronoid Process Hyperplasia or Langenbeck Disease: The Big Jumble. Comment on “Raccampo et al. Jacob’s Disease: Case Series, Extensive Literature Review and Classification Proposal. *J. Clin. Med.* 2023, *12*, 938”

**DOI:** 10.3390/jcm12155118

**Published:** 2023-08-04

**Authors:** Luca Raccampo, Giorgio Panozzo, Alessandro Tel, Lorenzo Trevisiol, Antonio D’agostino, Salvatore Sembronio, Massimo Robiony

**Affiliations:** 1 Maxillofacial Surgery Department, Academic Hospital of Udine, Department of Medicine, University of Udine, 33100 Udine, Italy; 2 Section of Oral and Maxillofacial Surgery, Department of Surgical Sciences, Dentistry, Gynaecology and Paediatrics, University of Verona, 37129 Verona, Italy

As authors of the text, we can only thank Mattei et al. [1]. for the opportunity to deepen some concepts regarding Jacob’s disease (JD) in response to our previous work in which we tried to summarize its main characteristics with some examples and a review of the literature.

They expressed concern regarding the definition of JD that we reported. In particular, the concepts of pseudo-joint and the involvement of a morphological change in the coronoid process were identified, which seems to be truly a big jumble deepening this topic. Their reply tries to better explain that the original definition of Jacob’s disease does not report any reference to an abnormal coronoid process in the mandible, stating as Octave Jacob described “a coronoid process of normal shape and size associated with a hyperostosis of the posterior part of the zygomatic bone [2]. The posterior surface of the zygomatic bone and the coronoid process was covered by cartilaginous tissue forming a pseudojoint”. This definition summarizes Jacob’s findings and the name of Jacob’s disease given to this condition by Chemin and Bercher in 1958 [3]. If you carefully read Jacob’s post-mortem description of the case, you will find out how he described the formation of a true artrodia between the coronoid process and the inner aspect of the ipsilateral malar bone. The two articular surfaces were reported as capped at the cartilage, with a true capsule formed by fibrous tissue and tendon fibers of the temporal muscle. Therefore, Jacob effectively described what the definition reported by Mattei et al. stands for. However, this description was not supported by any histological examination and was merely backed up by a macroscopic description of the specimens. Thus, a histologically proven pathological condition to the coronoid process or malar bone may better point out some characteristics of the pathology and the single case. What is stated for sure is the formation of a pseudo-joint, which Mattei et al. also seem to highlight and support. The original definition may be then considered incomplete or dated and limited to the formation of a pseudo-joint between the inner aspect of the malar bone and the coronoid process, without any specific reference to whether morphological abnormalities affected or not one or both joint surfaces. It is superfluous to specify that in order to make this diagnosis, a pathological event must involve either the coronoid process or the zygomatic bone or both; otherwise, the two bone segments would not have the anatomical basis to come into contact. The definition we reported tried to summarize what is specified in the literature, which, as we both reported, is not univocal and leads to a lot of confusion. Indeed, it could easily make any classification difficult [4]. That is why we tried to perform a review of the articles about this condition. Analyzing the 116 cases reported, we were able to confirm that there was not a unique etiological factor nor a unique histological diagnosis that framed this pathology. In fact, many authors theorize a different etiology, from a genetic or endocrine cause to traumas, TMJ disorder, and the idiopathic factor. Moreover, according to the different amounts and patterns of bone and cartilage tissue present, the histological examination of the specimens after the coronoidectomy can be really varied, progressing from the diagnosis of cartilage-capped exostosis (CCE) to coronoid hyperplasia (CH) with osteochondroma (OC) being the most common, among others [5,6,7,8,9,10,11]. We found 17 cases of CCE (14.7%), 10 cases of CH (8.6%), and 76 cases of OC (65.5%), while in 13 cases (11.2%), it was not possible to establish a definite histological diagnosis [4]. This marked prevalence of OC as a cause of JD could partly explain the confusion over its definition and its almost exclusive bi-directional association with pathology reported by various authors. As correctly specified by Mattei et al., JD is, therefore, limited to a pseudo-joint formation between the zygomatic bone and coronoid process, and this concept is clearly made explicit in our work. Another aspect to consider is the correct definition of the term “pseudojoint”. This term indicates an even greater pathological connection between two bony surfaces in which at least one can move in a certain direction. Over time, they begin to be covered by a thin layer of cartilage, and in the surrounding tissues, probably under an inflammatory stimulus, a sort of capsule gradually develops. The result is a non-functional articulation, which gradually allows less and less movement. The same mechanism can be observed in pseudo-arthrosis. Considering that this is a process that occurs over time, the histological connotation regarding the presence of cartilage on the articular surfaces can also be cleared. What is certain is that a chronic impingement between the two articular surfaces leads to a response from both, which may modify their morphology, and over time cause a pseudo-joint formation. Moreover, it could be difficult to analyze the surrounding tissues for the definition of a pseudo-capsule due to the surgical maneuver, which inevitably damages them, even if only partially. In these terms, thanks to Computed Tomography (CT), this disease should be recognized after a proper clinical evaluation and the exclusion of other more common local and systemic pathologies and, if possible, even with the support of a histological investigation. Overall, JD is rare and messy to be defined and diagnosed condition. A consensus is needed for its definition, and further studies are desirable to identify better methods for a univocal diagnosis of this disease.
